# Acute hepatitis-like presentation with cholestasis of *CBFB–MYH11*-positive acute myeloid leukemia in an adult male: a case report

**DOI:** 10.1186/s13256-022-03476-7

**Published:** 2022-07-31

**Authors:** Irene Spinelli, Adriano De Santis, Laura Cesini, Mara Riminucci, Alessandro Corsi, Mariana Forlino, Elio Pietro Perrone, Clara Minotti, Claudio Cartoni

**Affiliations:** 1 Department of Translational and Precision Medicine, Sapienza University, Viale dell’Università 37, 00185 Rome, Italy; 2 Department of Molecular Medicine, Sapienza University, Viale Regina Elena 324, 00161 Rome, Italy

**Keywords:** Leukemia, Hepatitis, Chemotherapy, Ultrasound, Case report

## Abstract

**Background:**

Liver involvement in adults with acute myeloid leukemia is uncommon. Most of the case reports describe acute liver failure or obstructive jaundice, while acute hepatitis is rarely mentioned. We report a patient with acute myeloid leukemia who presented with clinical, biochemical, and radiological signs of acute hepatitis that totally regressed after chemotherapy.

**Case presentation:**

A 38-year-old Caucasian man presented with fever, cough, and mild fatigue. Laboratory workup showed anemia, thrombocytopenia, severe leukocytosis, transaminitis, and hyperbilirubinemia. Imaging of the abdomen (ultrasound and magnetic resonance) showed hepatomegaly, splenomegaly, upper limits portal veins diameters, increased thickness of the gallbladder wall, and significant abdominal lymph nodes. Peripheral blood smear and bone marrow evaluation were consistent with acute myeloid leukemia, and liver biopsy showed massive sinusoidal and portal infiltration by leukemic cells. After remission-inducing chemotherapy, there was complete normalization of liver function tests, and liver, spleen, and portal vein size.

**Conclusions:**

This case highlights the importance of taking acute myeloid leukemia into account as a possible cause of liver damage to make a rapid diagnosis and start appropriate treatment that may lead to hematological remission and hepatic dysfunction resolution.

## Introduction

Liver involvement in adults with acute myeloid leukemia (AML) is uncommon. Most of the case reports describe acute liver failure or obstructive jaundice, while acute hepatitis is rarely mentioned. We report a patient with AML who presented with clinical, biochemical, and radiological signs of acute hepatitis that totally regressed after chemotherapy.

## Case presentation

A 38-year-old Caucasian man presented with fever, cough, and mild fatigue with a 10-day duration. Past medical history was remarkable for hepatic steatosis, without abnormal liver tests but with hypercholesterolemia in treatment with statins, and negative for alcohol consumption. Physical examination showed jaundice and hepatosplenomegaly; body mass index (BMI) was 29.4 kg/m^2^.

Laboratory results are presented in Table 1. Serum electrolytes, renal function, and blood gases were normal. Test for Epstein–Barr virus (EBV), cytomegalovirus (CMV), hepatitis A, B, and C virus, and autoimmune workup were negative.

**Table 1.** Laboratory results

AST (UI/L)	163	Hb (g/dL)	7.3
ALT (UI/L)	347	WBC (× 10^3^/µL)	115
Alkaline phosphatase (UI/L)	463	Neutrophils (%)	66
GGT (UI/L)	733	Monocytes (%)	23
Bilirubin (mg/dL)	8.98	Lymphocytes (%)	3
Conjugated bilirubin (mg/dL)	8.35	Eosinophils (%)	5
Albumin (g/dL)	3.5	Basophils (%)	2
Glucose (mg/dL)	167	PLTS (× 10^3^/µL)	25
Uric acid (mg/dL)	10.3	Prothrombin time (s)	11.4
LDH (UI/L)	1323	INR	1.26

Normal laboratory values are as follows: AST 8–38 UI/L, ALT 12–41 UI/L, alkaline phosphatase 40–129 UI/L, GGT 8–61 UI/L, bilirubin 0.35–1 mg/dL, conjugated bilirubin 0.15–0.35 mg/dL, albumin 3.5–5.2 g/dL, glucose 74–106 mg/dL, uric acid 3.4–7 mg/dL, LDH 135–225 UI/L, hemoglobin 13–17 g/dL, white blood cells 4–10 × 10^3^/µL, neutrophils 40–74%, monocytes 3.4–11%, lymphocytes 19–48%, eosinophils 0–7%, basophils 0–2%, platelets 150–450 ×10^3^/µL, prothrombin time 7.87–10.15 seconds. *AST* aspartate aminotransferase, *ALT* alanine aminotransferase, *GGT* gamma-glutamyl transpeptidase, *LDH* lactate dehydrogenase, *Hb* hemoglobin, *WBC* white blood cells, *PLTS* platelets, *INR* internationalized ratio

**Fig. 1:**
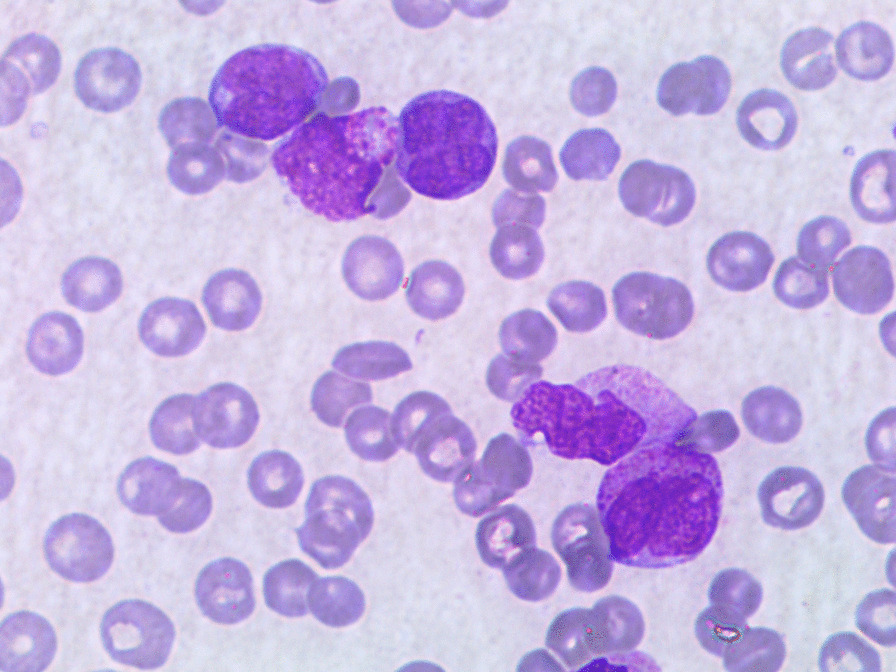
Peripheral blood smear at diagnosis

**Fig. 2:**
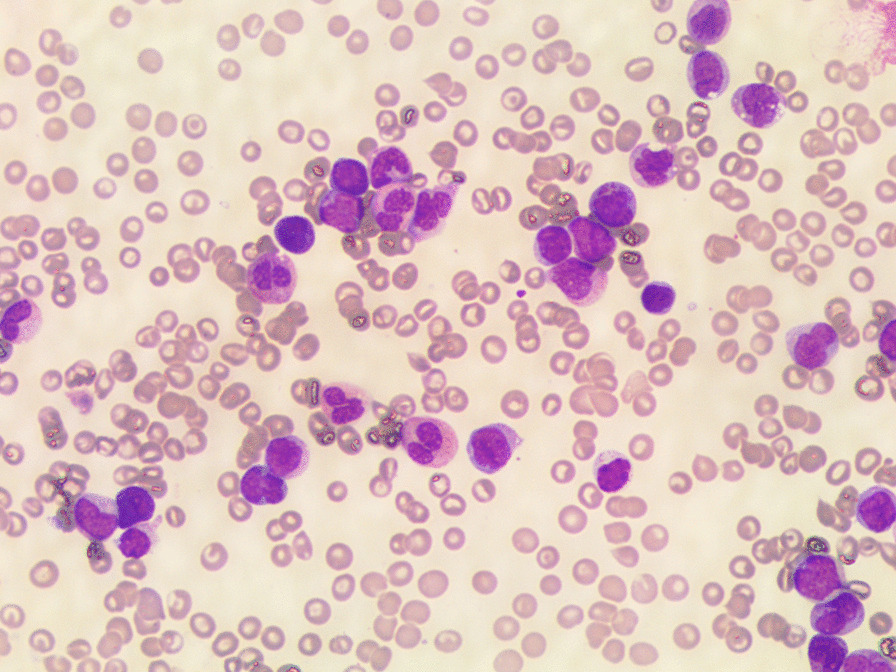
Peripheral blood smear at diagnosis

**Fig. 3:**
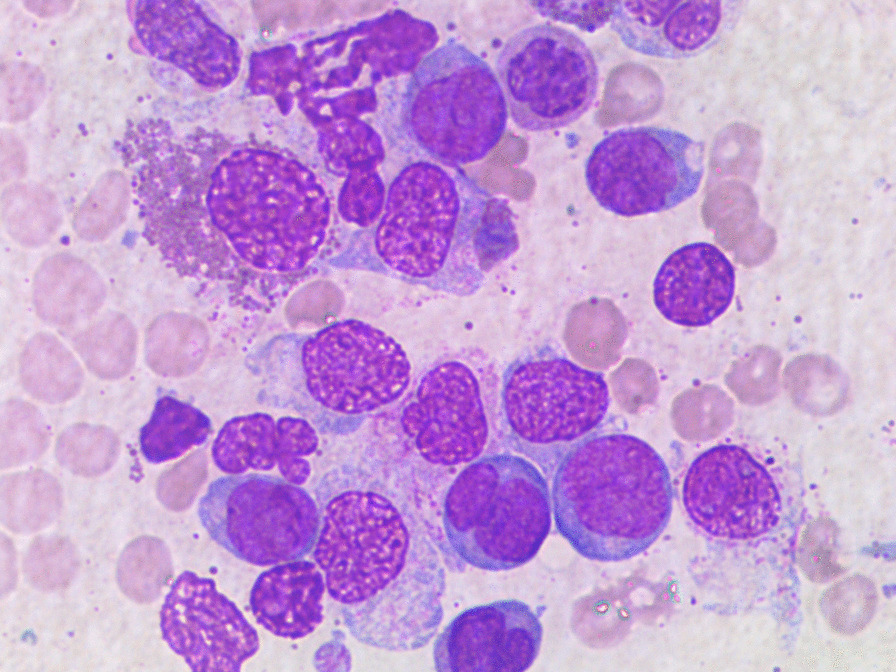
Bone marrow aspiration at diagnosis

**Fig. 4:**
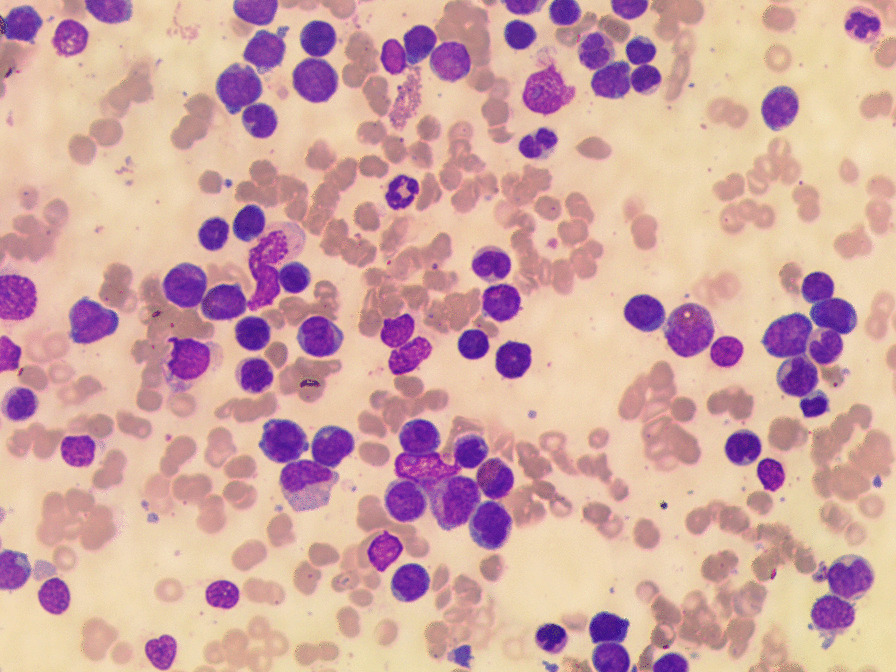
Bone marrow aspiration at diagnosis

Peripheral blood (PS) smear (Figs. 1, 2) analysis showed 15% blasts, therefore he was hospitalized and underwent a bone marrow (BM) aspiration (Figs. 3 and 4).

The cytomorphologic evaluation of BM revealed increased cellularity, 40% blasts positive to myeloperoxidase (MPO), with Auer rods, a picture compatible with AML.

The flow cytometry immunophenotyping of the BM sample revealed 54% of myeloid blasts (CD34^+/−^/CD117^+/−^/ CD133^+/−^/CD38^+^/CD11b^+^/ CD4^+^/CD33^+^/CD14^+^/ CD13^+^/HLA-DR^+^), with MPO expressed on 80% of leukemic cells.

Conventional cytogenetic analysis was attempted but yielded no metaphase cells for analysis, while molecular analysis, performed using real-time polymerase chain reaction (RT-PCR), showed the presence of the *CBFB–MYH11* fusion gene.

**Fig. 5:**
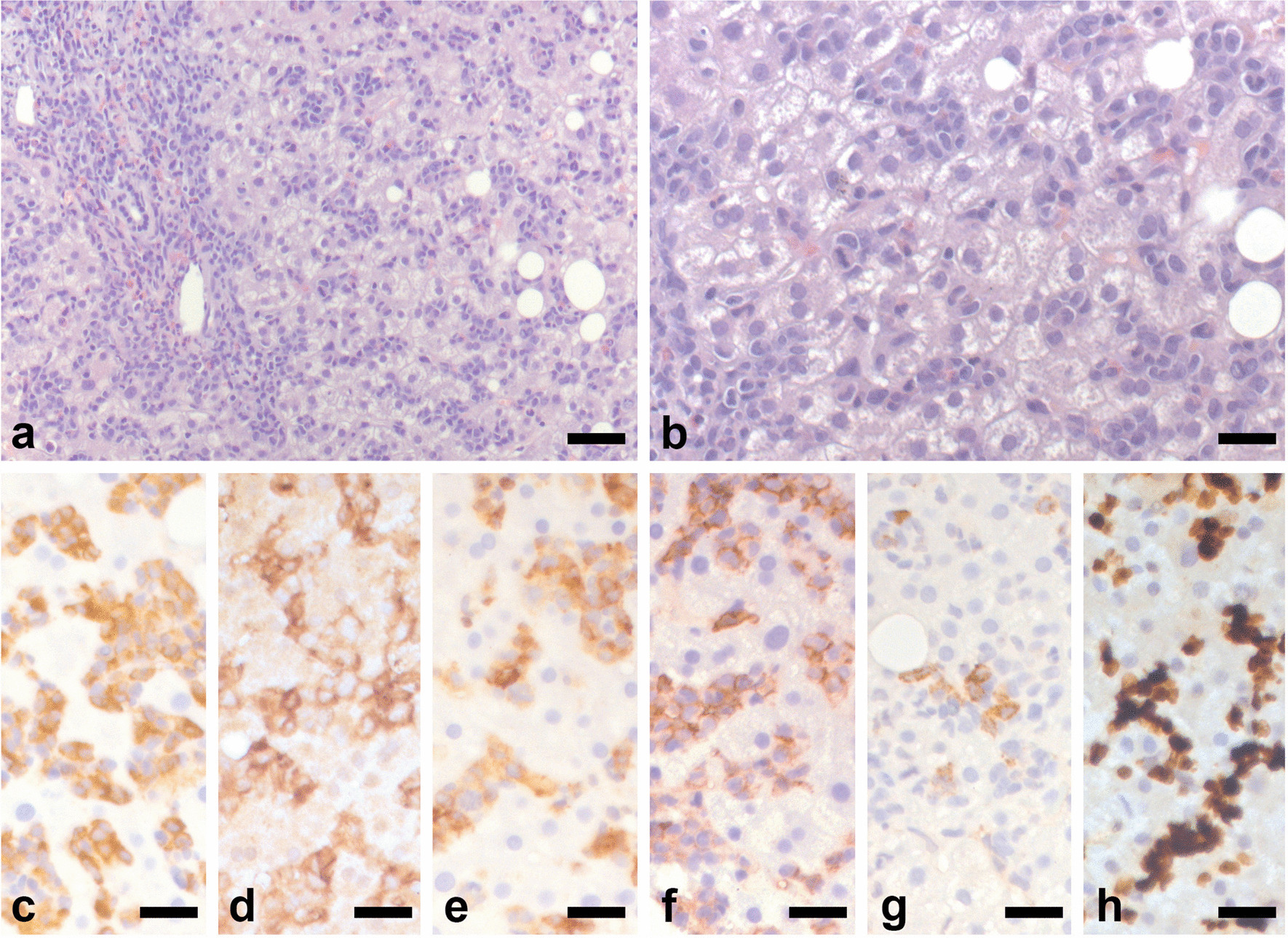
Histological representative images of the liver biopsy shown in **a** and **b**. Leukemic cells infiltrate the portal tract and the sinusoids. Please note the abundance of eosinophils, in particular within the portal tract. Panels **c**–**h** illustrate the immunoreactivity of the leukemic cells for MPO (**c**), CD33 (**d**), HLA-DR (**e**), CD38 (**f**), CD34 (**g**), and Ki67 (**h**). Bars: 100 μm in **a** and 50 µm in **b**–**h**.

Abdominal ultrasonography examination described hepatomegaly (longitudinal diameter of right lobe 19.5 cm), splenomegaly (longitudinal diameter 18 cm), upper limits portal vein diameters (portal vein 13 mm, splenic vein 9.2 mm, superior mesenteric vein 9.5 mm), normal portal vein mean velocity (21.5 cm/second), increased thickness of the gallbladder wall (9 mm), enlarged abdominal lymph nodes (maximum diameter 20.9 mm), normal biliary ducts and a liver stiffness value of 8.16 kPa using acoustic radiation force impulse (ARFI) (Table 2). Abdominal magnetic resonance confirmed the dimensional findings and excluded biliary tree dilatation. In order to explain the cause of abnormal liver tests and to exclude preexisting liver disease, a liver biopsy was performed. It showed massive sinusoidal and portal infiltration by leukemic cells (Fig. 5a, b). Many eosinophils were also observed. Leukemic cells were immunoreactive for MPO (Fig. 5c), CD33 (Fig. 5d), HLA-DR (Fig. 5e), CD38 (Fig. 5f), and, in part, for CD34 (Fig. 5g). Virtually all blasts expressed Ki67 (Fig. 5h). Additional features consisted of cholestasis, macrovacuolar steatosis and perisinusoidal fibrosis equal to stage 2 on the Ishak Fibrosis Score (fibrous expansion of most portal areas, with or without short fibrous septa).

**Table 2:** Abdominal organ diameters by ultrasound scan

Abdominal organ	Before induction chemotherapy	After induction chemotherapy	After two consolidation chemotherapy cycles
Liver (cm)	19.5	14.5	14.5
Spleen (cm)	18	14	13.3
Portal vein (mm)	12	Normal	9.7
Splenic vein (mm)	9.2	Normal	7
Superior mesenteric vein (mm)	9.5	Normal	7.8
Portal vein mean velocity (cm/second)	21.5	Not available	23
ARFI (kPa)	8.16	Not available	3.96
Gallbladder wall (mm)	9	Normal	< 4
Abdominal lymph nodes (max. diameter, mm)	20.9	Not available	Not visible

*cm* centimeters,* mm* millimeters,* cm/s* centimeters/seconds,* Kpa* kilopascal

The patient started intensive front-line induction therapy according to “3 + 7” regimen with reduced doses of antracycline to limit hepatic toxicity [daunorubicine 45 mg/m^2^, days 1–3 plus citarabine (Ara-c) 100 mg/m^2^ continuous infusion (CI) days 1–7].

We observed a progressive reduction of the values of AST, ALT, alkaline phosphatase, and bilirubin, and a complete normalization was obtained on day +15 from the beginning of chemotherapy. Lactate dehydrogenase (LDH) became normal too.

After induction therapy, abdominal ultrasonography examination was performed, revealing reduction of abdominal organ sizes. The liver and spleen diameter was 14.5 and 14 cm, respectively, normal portal vein diameters and thickness of the gallbladder wall, not evaluated portal vein mean velocity, abdominal lymph nodes diameters, and liver stiffness value. The ultrasound scan was repeated after two consolidation chemotherapy cycles and it showed complete normalization of all parameters, an event that was not mentioned in the previous study but now evaluated (Table 2).

After induction, the BM evaluation showed a morphological complete remission (CR) of AML, but the persistence of leukemia at the molecular biology analysis, with a minimal residual disease (MRD) of 39.19 copies (cp)/10^4^ ABL cp.

After induction, the patient received two consolidation chemotherapy courses: the first with daunorubicine 50 mg/m^2^ (days 5–6) plus Ara-c 500 mg/m^2^ bid (days 1–6) and the second with high doses of Ara-c 2 g/m^2^ bid (days 1, 3, 5). Molecular biology examination confirmed the MRD persistence after both consolidation cycles (66 and 42.62 cp/10^4^ ABL cp, respectively); therefore, the patient was considered eligible for allogenic stem cell transplantation (allo-SCT).

He received conditioning chemotherapy with Thiothepa Busulfan Fludarabine (TBF) scheme (Thiotepa 5 mg/kg/, on days 7 and 6; Busulfan 3.2 mg/kg/day, and Fludarabine 50 mg/m^2^/day from day 5 to day 3), followed by allogenic stem cells infusion from sibling donor on day 0.

The BM evaluation on day +20 post-SCT showed a morphologic CR with complete chimerism. During allo-SCT hospitalization, he developed febrile neutropenia responsive to antibiotic therapy. No hepatic complications were observed. The patient was discharged from the hospital and started standard follow-up.

## Discussion and conclusions

The reported case describes AML infiltrating the liver in a young male adult. Among different subtypes of AMLs, *CBFB–MYH11* AML usually has a component of monoblastic/monocytic differentiation, a phenotype that may be associated with myeloid sarcoma (MS). MS may arise before the presentation of, concurrently with, or as a relapse of AML. Diagnosis is largely dependent on tissue biopsy, which mostly consists of infiltration by myeloid leukemic cells. Immunohistochemistry, flow cytometry, and molecular analysis further help with definitive diagnosis. With concomitant bone marrow involvement, the more common extramedullary sites of leukemic cell infiltration include the skin and gingiva. On the other hand, MSs commonly involve the bone, lymph nodes, soft tissue, gastrointestinal tract, mediastinum, and gonads [1].

Typically, MS with *CBFB–MYH11* fusion has the anatomic predilection for abdominal locations. The gastrointestinal tract is frequently involved, particularly the small intestine. Although MS can develop in any organ, liver infiltration is very rare, therefore it presents as a diagnostic challenge, especially in patients with no previous history of myeloproliferative neoplasm or acute leukemia, as happened in our patient [2, 3].

Bone marrow infiltration and liver involvement were documented simultaneously in our case; Furthermore, no previous hepatic disease was notified in his medical history so we could not assess whether MS preceded or followed AML. Liver biopsy was performed to confirm AML localization and to exclude hepatobiliary malignancies.

Regardless of bone marrow involvement, MS should be considered as AML and treated as such. The recommended regimen is systemic chemotherapy using AML-induction protocols; surgery can be used for debulking before starting chemotherapy. The decision to proceed with allogeneic stem cell transplant as opposed to conventional chemotherapy is mostly based on the clinical features suggestive of aggressive disease. In our case we treated the patient according to AML protocols, obtaining a prompted resolution of biochemical results, abdominal organ sizes, and medullary infiltration. Considering the aggressive presentation of AML at the diagnosis, and the MRD persistence after consolidation therapy, we decided to perform allogenic stem cell transplant, obtaining a complete response.

The other point of interest of the case is the hepatic involvement in AML. It is in fact very rare in adults and is more frequent in children and in the setting of acute lymphoblastic leukemia [4–12]. In addition, the majority of infiltrating liver AML cases present as acute liver failure or obstructive jaundice [13]. Conversely, here we mention a hepatitis-like picture with raised serum aminotransferase, gamma-glutamyl transpeptidase, alkaline phosphatase, and bilirubin levels, but normal prothrombin time. The patient’s mental status was normal, and there were no signs of hepatic encephalopathy.

The causative mechanism of liver injury is due to sinusoid infiltration by leukemic cells that induces tissue ischemia, with raised transaminase levels, and progresses to necrosis, presenting as liver failure [14, 15]. Another consequence of liver damage is hepatic clearance reduction, which leads to hyperlactatemia. Lactate production by tumor cells and hypoxic tissues may also contribute to its increase [16, 17].

Although acute leukemia is an uncommon cause of liver injury, presenting as acute liver failure, obstructive jaundice, or acute hepatitis, our report, as well as previous ones, underline that it should be considered as possible etiology [8, 18, 19]. Therefore, it is important to pay attention to patients with prodromal symptoms and abnormal hematological and biochemical analyses, such as anemia, neutropenia, thrombocytopenia and raised white cell count, lactate, uric acid, and LDH (the last two being indicators of high cell turnover). In suspected cases, PB smear and BM examination are mandatory [20].

Furthermore, the alteration of liver biochemical results does not necessarily reflect a primitive liver disease. In our case, there is another confounding factor besides elevated hepatic tests, viz. abnormal abdominal findings on imaging. Abdominal ultrasound revealed hepatosplenomegaly, upper limits portal vein diameters, and mild-range liver stiffness value (8.16 kPa using ARFI). Thus, the first two parameters mentioned above are portal hypertension hallmarks and disagree with the liver stiffness, which was not too elevated to be diagnostic for cirrhosis. The association between abnormal clinical, biochemical, and radiological liver parameters may have simulated a chronic liver disease, but this was excluded by the liver stiffness. In this setting, liver biopsy was indicated to confirm the hypothesis of liver involvement by AML and exclude preexisting hepatic disease. It has also been confirmed by rapid and complete regression of both biochemical results and abdominal organ sizes during chemotherapy to underline that liver condition is the consequence of hematological malignancy [19, 21, 22].

This case report highlights the importance of taking AML into account as a possible cause of liver damage, which could evolve to liver failure, to make a rapid diagnosis and start appropriate treatment. Although early recognition is essential, in most cases this condition presents poor prognosis and cannot be resolved by liver transplantation [23, 24]. On the contrary, chemotherapy may lead to hematological remission and hepatic dysfunction resolution, as occurred in our patient.

## Data Availability

The datasets used and/or analyzed during the current study are available from the corresponding author on reasonable request.
